# JMJD5 links CRY1 function and proteasomal degradation

**DOI:** 10.1371/journal.pbio.2006145

**Published:** 2018-11-30

**Authors:** Anand R. Saran, Diana Kalinowska, Sangphil Oh, Ralf Janknecht, Luciano DiTacchio

**Affiliations:** 1 Department of Pharmacology, Toxicology, and Therapeutics, University of Kansas Medical Center, Kansas City, Kansas, United States of America; 2 Department of Cell Biology, University of Oklahoma Health Sciences Center, Oklahoma City, Oklahoma, United States of America; Charité Universitätsmedizin Berlin, Germany

## Abstract

The circadian oscillator is a molecular feedback circuit whose orchestration involves posttranslational control of the activity and protein levels of its components. Although controlled proteolysis of circadian proteins is critical for oscillator function, our understanding of the underlying mechanisms remains incomplete. Here, we report that JmjC domain–containing protein 5 (JMJD5) interacts with CRYPTOCHROME 1 (CRY1) in an F-box/leucine-rich repeat protein 3 (FBXL3)-dependent manner and facilitates targeting of CRY1 to the proteasome. Genetic deletion of JMJD5 results in greater CRY1 stability, reduced CRY1 association with the proteasome, and disruption of circadian gene expression. We also report that in the absence of JMJD5, AMP-regulated protein kinase (AMPK)-induced CRY1 degradation is impaired, establishing JMJD5 as a key player in this mechanism. JMJD5 cooperates with CRY1 to repress circadian locomotor output cycles protein kaput (CLOCK)–brain and muscle ARNT-like protein 1 (BMAL1), thus linking CRY1 destabilization to repressive function. Finally, we find that ablation of JMJD5 impacts FBXL3- and CRY1-related functions beyond the oscillator.

## Author summary

In mammals, circadian rhythms are generated by a molecular oscillator in which the circadian locomotor output cycles protein kaput (CLOCK)–brain and muscle ARNT-like protein 1 (BMAL1) transcription factors drive expression of the genes coding for their own repressors, the CRYPTOCHROME (CRY) and PERIOD (PER) proteins. A key feature of the oscillator is that the protein stability of its components is highly regulated. Previous studies had implicated the JmjC domain–containing protein 5 (JMJD5) in regulation of the circadian clock in plants and flies. Here, we show that cells and livers that lack JMJD5 exhibit dysregulation of circadian gene expression. Mechanistically, JMJD5 is required for CRY1 degradation, including its destabilization by AMP-regulated protein kinase (AMPK), by facilitating its interaction with the proteasome. We found that JMJD5 is needed for normal CRY1-mediated transcriptional repression, thereby uncovering an inverse relationship between CRY1 stability and circadian repression. Finally, we showed that JMJD5 impinges on non-clock roles of F-box/leucine-rich repeat protein 3 (FBXL3) and CRY1. Altogether, our studies demonstrate that JMJD5 is a novel link between the oscillator and other physiological processes.

## Introduction

Circadian rhythms are endogenous, approximately 24-hour oscillations in behavior and physiology that evolved as an adaptation to the day–night cycle. These rhythms are generated by a cell-autonomous timekeeping mechanism known as the molecular circadian oscillator. At its most basic, the oscillator is a transcription–translation circuit formed by two interlocked delayed negative feedback loops [1]. In one loop, the transcription factors circadian locomotor output cycles protein kaput (CLOCK) and brain and muscle ARNT-like protein 1 (BMAL1) drive expression of the genes coding for their own repressors, the CRYPTOCHROME (CRY) and PERIOD (PER) proteins, leading to alternative cycles of transcription activation and repression—the molecular basis of the clock. In a second loop, the opposing actions of REV-ERB and ROR nuclear hormone receptors (NHRs) generate strong oscillations in *Bmal1* gene transcription, which contributes to robust amplitude in circadian rhythms. However, the function of the circadian oscillator involves a much larger repertoire of factors that include other transcription regulators, kinases, phosphatases, ubiquitin ligases and peptidases, and chromatin regulators. Together, this large cohort of molecules acts in concert to generate circadian rhythms, coordinate the clock with other physiological processes, and enable environmental information to be integrated into its function.

A key mode by which circadian rhythms are generated and fine-tuned is by the regulation of the protein levels of the core oscillator components [2]. For instance, phosphorylation of PER proteins by Casein kinase I (CKI) decreases their stability by stimulating their interaction with and ubiquitylation by the Skip-Cullin-F box (SCF)^β-TRCP1/2^ ubiquitin ligase complex [3,4]. Similarly, degradation of REV-ERBα by Homologous to the E6-AP Carboxyl Terminus (HECT)-(ARF-BP1) and Really Interesting New Gene (RING)-class E3 ligases (PAM) is induced by phosphorylation by glycogen synthase kinase 3β (GSK3β) [5,6]. BMAL1 is also phosphorylated by GSK3β, leading to its destabilization [7]. Although BMAL1 ubiquitylation has been found to be catalyzed by the HECT-class E3 ligase UBE3A [8], a link between this process and GSK3β-mediated phosphorylation has not been found. Taken together, these observations show that, although the mechanisms that control the stability of the clock proteins are similar, involving coordinated phosphorylation and ubiquitylation, there is divergence in the machinery that targets different components.

In mammals, CRY degradation is mediated by SCF ubiquitin ligase complexes that contain one of two closely related F-box/leucine-rich repeat proteins (FBXLs), FBXL3 and FBXL21 [9–13]. Although the two ligases can both ubiquitylate CRYs, their actions are antagonistic. In the nucleus, FBXL3 promotes K48-linked polyubiquitylation of CRYs, leading to its degradation, whereas FBXL21 binds with greater affinity to CRY yet catalyzes K48 polyubiquitylation less efficiently than FBXL3 [13]. Thus, presence of FBXL21 diminishes the overall CRY degradation. Despite its presence in the nucleus, FBXL21 localizes primarily to the cytoplasm, where it promotes CRY degradation, highlighting the complexity of CRY1 regulation. As with other clock proteins, CRY1 degradation is controlled by phosphorylation, most notably by the AMP-regulated protein kinase (AMPK) [14]. AMPK-mediated phosphorylation of CRY1 strengthens interactions with FBXL3, thereby leading to CRY destabilization. Yet, despite the fact that both mammalian CRY paralogs largely share the same degradation machinery, differences in how their levels are controlled appear to exist [15–17].

Members of the JmjC domain–containing family of proteins are characterized by a cupin-type domain of about 150 amino acids, known as the JmjC domain, which is able to confer lysine demethylase activity to some but not all proteins that harbor it [18]. In recent times, members of the JmjC family have emerged as important regulators involved in a variety of physiological processes, including control of circadian rhythms in plant, mammalian, and insect systems [19–22]. Previously, a genetic study identified *Arabidopsis thaliana Jmjd5* (*AtJmjd5*) as a regulator of the circadian system in plants that exhibits sufficient functional conservation with its mammalian ortholog JmjC domain–containing protein 5 (JMJD5) as to exhibit reciprocal rescue of circadian phenotypes arising from genetic ablation in plants or small interfering RNA (siRNA) knockdown in U2OS cells [19]. Similarly, in *Drosophila*, genetic deletion of JMJD5 leads to reduced period length in locomotor activity and decreased sleep [23]. However, though these studies firmly establish JMJD5 as an evolutionarily conserved participant of the clock, its mechanism of action in the clock has remained undefined.

Although JMJD5 has been suggested to be a lysine demethylase, such a function remains highly debated and is not yet firmly established [24–26]. Nonetheless, JMJD5 has been reported to influence gene transcription through several mechanisms, including modulation of protein levels, nuclear entry of transcription factors, and proteolytic processing of histone subunits [25,27–30]. We found that JMJD5 is recruited to CRY1–FBXL3 complexes, in which it facilitates CRY1 interaction with the proteasome. Furthermore, we report that JMJD5-dependent CRY1 destabilization is intertwined with the repressive function of CRY1.

## Results

### JMJD5 ablation disrupts circadian gene expression in fibroblasts and liver

**Fig 1:**
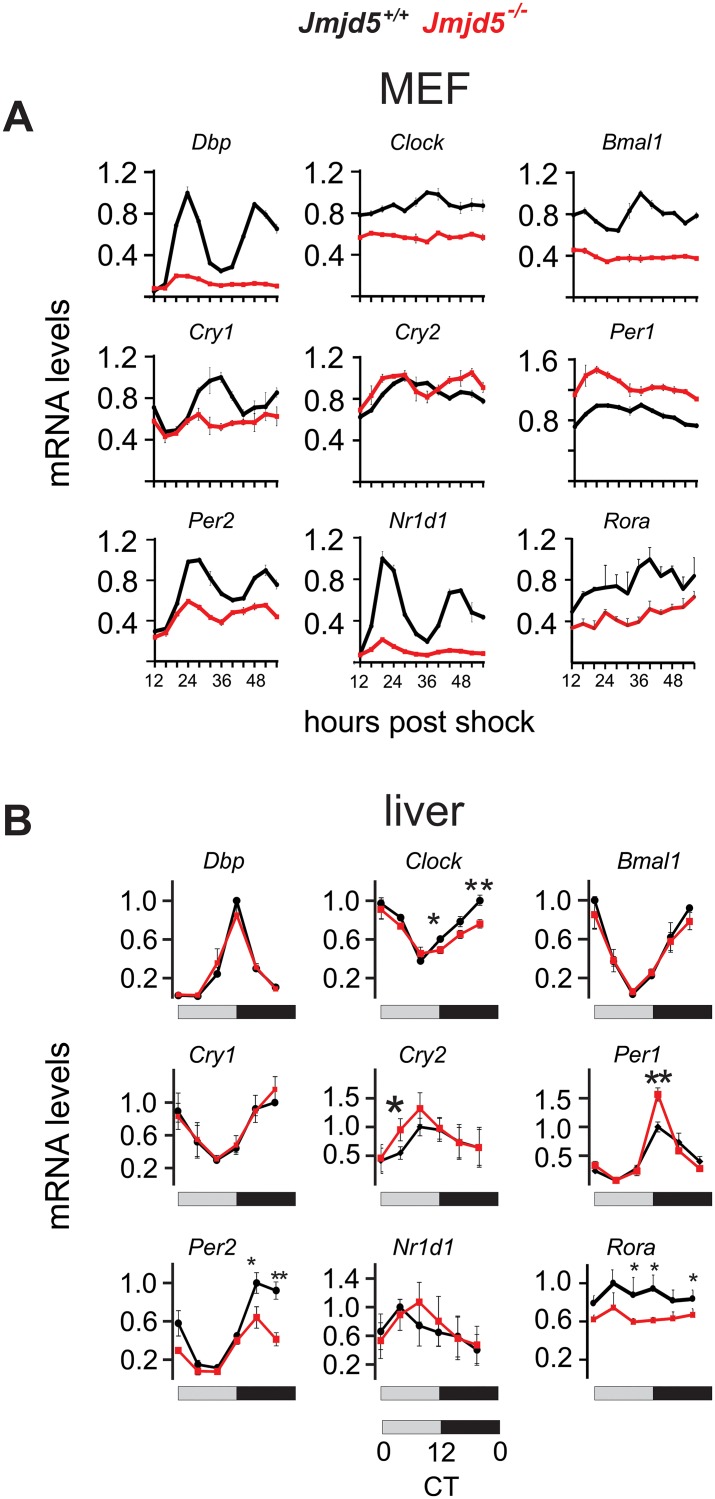
JMJD5 is a regulator of circadian gene expression. Genetic ablation of JMJD5 in (A) MEFs (mean ± SEM, *n* = 3) or (B) mouse liver disrupts the expression pattern and levels of clock genes (*p < 0.05, **p < 0.005 mean ± SEM, *n* = 4, one-tailed permutation test). Levels were normalized to *mrpl46*, and the maximum level of the indicated transcript in the wild-type genotype was set as 1. CT, circadian time; JMJD5, JmjC domain–containing protein 5; MEF, mouse embryo fibroblast.

To determine whether JMJD5 plays a role in the mammalian oscillator, we first analyzed the impact of its deletion on the circadian clock of mouse embryo fibroblasts (MEFs). We measured gene expression levels of core circadian oscillator components in a circadian timeline from *Jmjd5+/+* and *Jmjd5−/−* MEFs harvested at 4-hour intervals from 12 to 56 hours post synchronization with dexamethasone (Fig 1A and S1 Fig).

Cells that lack JMJD5 exhibit marked down-regulation of *Clock* and *Bmal1* mRNAs, the two central circadian transactivators. Consistent with a decrease in CLOCK–BMAL1 activity, mRNAs of their regulatory targets *Dbp*, *Cry1*, *Nr1d1*, and *Rora* showed similar decreases. In contrast, the impact of JMJD5 deficiency on *Cry2*, *Per1*, *and Per2* gene expression was divergent, with decreased *Per2*, unaffected *Cry2*, and increased *Per1* mRNA abundances. Real-time bioluminescence measurements from a *Per2* promoter-luciferase reporter also showed circadian dysfunction (S2 Fig), as *Jmjd5*-null cells exhibited shortened period length and decreased amplitudes in their oscillation, which not only confirmed our gene expression observations but also were consistent with previous reports [19,23].

Next, we assessed the impact of JMJD5 on circadian clock gene expression in vivo. Full-body *Jmjd5*-null mutant mice are embryonic lethal [26], but hepatocyte-specific *Jmjd5*-ablated animals (*Jmjd5* liver knockouts [*Jmjd5*^*LKO*^]) are viable and exhibit no overt phenotype. We generated a circadian liver timeline from wild-type and *Jmjd5*^*LKO*^ animals at a 4-hour resolution. As observed in fibroblasts, JMJD5 deficiency disrupts clock gene expression in a similar albeit nonidentical manner. Specifically, in both cells and liver that lack JMJD5, *Per1* mRNA was increased, and those of *Clock*, *Per2*, and *Rora* were decreased (Fig 1A and 1B). In contrast to fibroblasts, JMJD5-null livers showed no defect in the expression patterns of *Dbp*, *Bmal1*, *Cry1*, and *Nr1d1* mRNA (Fig 1B and S1 Fig).

**Fig 2:**
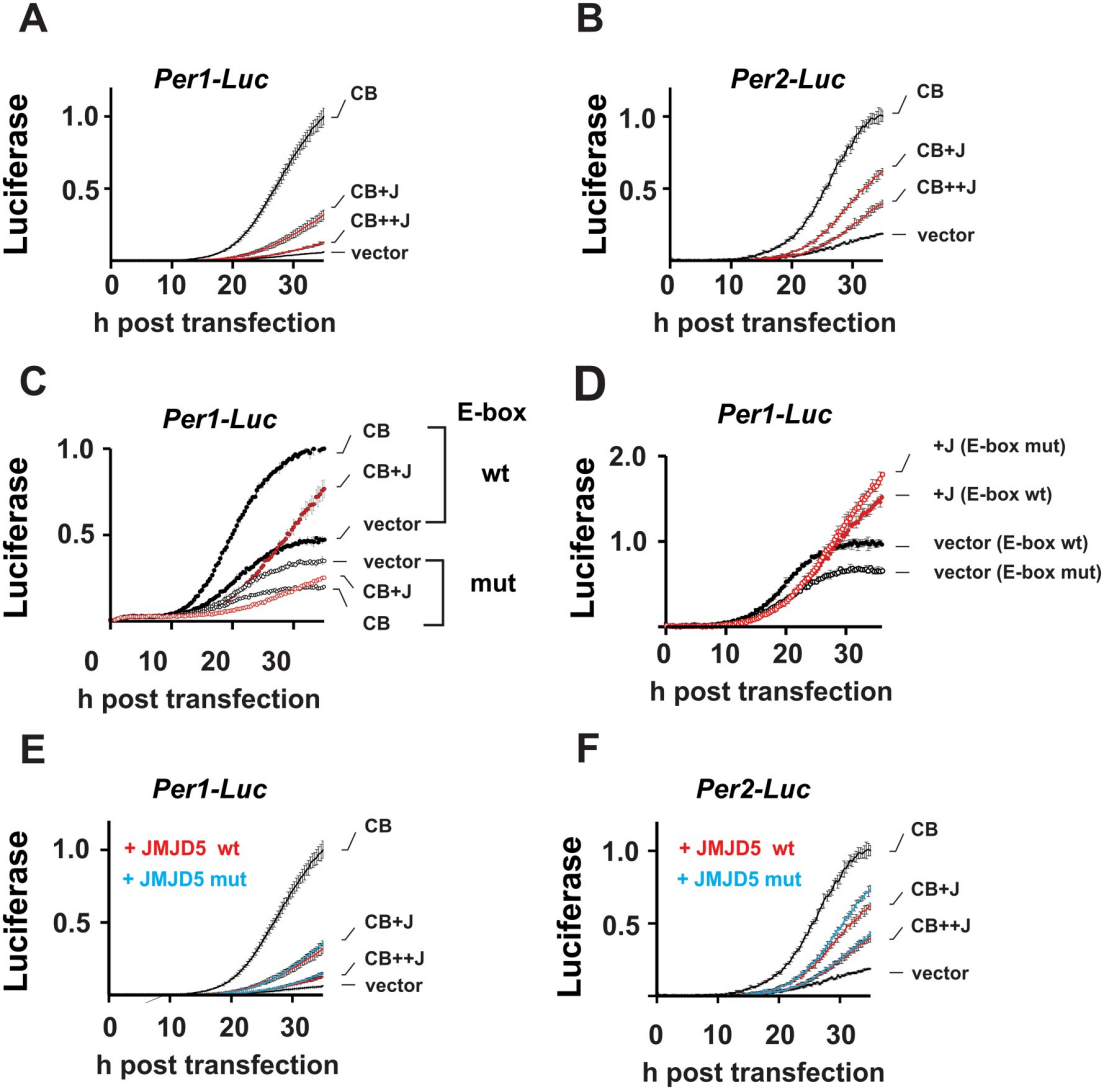
JMJD5 is a repressor of the circadian clock. (A, B) Dose-response repression of CLOCK and BMAL1 (“CB”) by JMJD5 (“J”). Real-time luciferase measurements of *Per1* and *Per2* promoter activity in non-oscillating HEK293T cells show repression of CB by JMJD5. Shown: counts normalized to maximal CB activation of the indicated reporter (mean ± SD) (C) JMJD5 repression is E-box mediated. JMJD5 represses *Per1-Luc* with a wt but not one with a mutant (“mut”) E-box (mean ± SD). (D) JMJD5 effect on wt and E-box mutant *Per1* reporters in the absence of CB. Shown: counts normalized to maximum signal obtained with wt reporter in the absence of JMJD5. (E) *Per1* and (F) *Per2* promoter is independent of the catalytic activity of JMJD5, normalizations performed as in A (mean ± SD). Please note that data plotted in A and E and in B and F were collected simultaneously; V, CB, and CB+JMJD5 in E and F are the same data as in A and B, respectively. BMAL1, brain and muscle ARNT-like protein 1; CLOCK, circadian locomotor output cycles protein kaput; HEK293T, human embryonic kidney 293T; JMDJ5, JmjC domain–containing protein 5; V, vector; wt, wild type.

Next, we assessed the impact of JMJD5 on CLOCK–BMAL1-mediated transcription in a series of real-time luciferase-reporter assays performed in the non-oscillating human embryonic kidney 293T (HEK293T) cell line. When coexpressed, JMJD5 decreased CLOCK–BMAL1 activation from *Per1-* and *Per2*-driven promoter-driven luciferase reporters in a dose-response manner (Fig 2A and 2B).

Repression of CLOCK–BMAL1 by JMJD5 is dependent on the presence of a functional E-box (Fig 2C). We observed that in the E-box mutant promoter, CLOCK–BMAL1 had a suppressive effect; it is possible that this effect is due to sequestration of limiting factors by CLOCK–BMAL1 away from the mutant promoter construct. Further, inclusion of JMJD5 with CLOCK–BMAL1 did not change this effect. In the absence of CLOCK–BMAL1, JMJD5 had only a minor repressive effect on the wild-type *Per1* promoter and no such effect in the E-box mutant construct, demonstrating that CLOCK–BMAL1-mediated activation is required for JMJD5 repression (Fig 2D). Although disputed, JMJD5 has been suggested to possess catalytic activity by virtue of its JmjC domain. To define whether any such activity is necessary for its effect on CLOCK–BMAL1 activity, we assessed CLOCK–BMAL1 repression by JMJD5^H321A^, a mutant construct that harbors a mutation in a conserved residue required for cofactor binding by the JmjC domain, thus precluding any enzymatic function [30–32]. JMJD5^H321A^ repressed CLOCK–BMAL1 activation of both *Per1*- and *Per2*-luciferase reporter constructs, indicating that the circadian function of JMJD5 does not require catalytic activity (Fig 2E and 2F). The only other two JmjC proteins that have been shown to participate in the mammalian clock—JARID1A and FBXL11/lysine-specific demethylase 2A (KDM2A)—also do so in a catalytically independent manner [21,22].

### JMJD5 destabilizes CRY1

**Fig 3:**
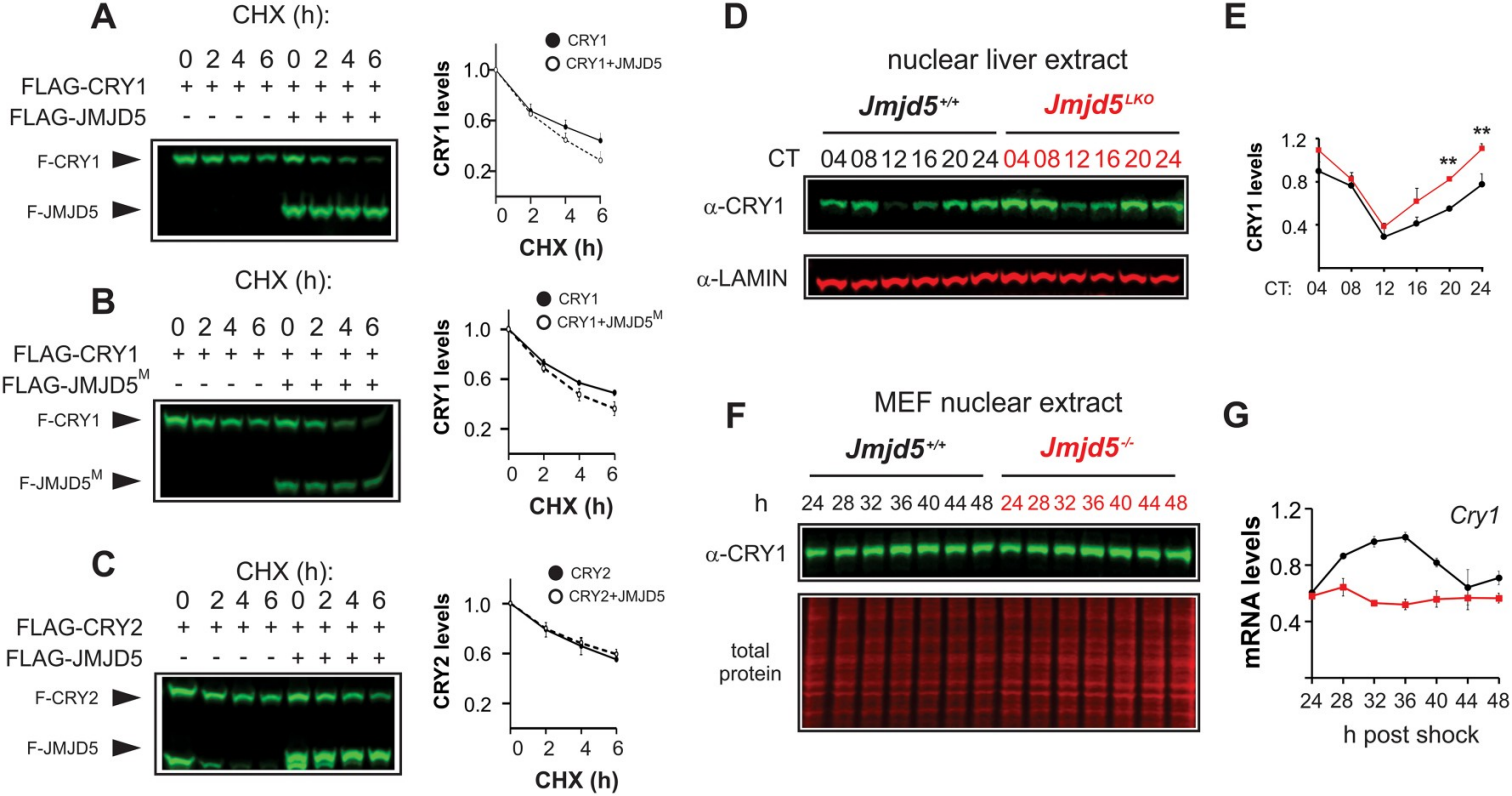
JMJD5 destabilizes CRY1. Impact of (A) wild-type and (B) catalytically inactive JMJD5^M^ on CRY1 stability. (C) JMJD5 is unable to degrade FLAG-CRY2. Densitometric analysis of independent experiments for A–C are shown next to the corresponding blots (mean ± SEM, *n* = 4, *n* = 3, and *n* = 3 respectively). Please note the presence of an unknown lower molecular weight band observed in FLAG-CRY2 transfections. (D) JMJD5-null livers have increased levels of endogenous nuclear CRY1 protein across the circadian cycle. (E) Densitometric analysis of CRY1 levels in control and JMJD5-null livers corresponding to (D) (mean ± SEM, *n* = 3). (F) CRY1 levels in nuclear extracts of *Jmjd5+/+* and *Jmjd5−/−* MEFs. *Cry1* mRNA levels from Fig 1 have been replotted to span 24–48 hours post synchronization. CHX, cycloheximide; CRY, CRYPTOCHROME; JMDJ5, JmjC domain–containing protein 5; *Jmjd5*^*LKO*^, *Jmjd5* liver knockout; JMJD5^M^, JMJD5 mutant; MEF, mouse embryo fibroblast.

JMJD5 has previously been reported to influence other transcription factors via regulation of their stability [25]. Thus, we performed cycloheximide chase assays to assess whether JMJD5-mediated repression of CLOCK–BMAL1 was due to induction of their degradation. JMJD5 did not influence CLOCK or BMAL1 degradation but instead markedly destabilized CRY1 in a catalytically independent manner (Fig 3A and 3B).

In contrast, JMJD5 had no effect on other clock proteins, including CRY2 (Fig 3C and S5 Fig). Consistent with our cycloheximide assays, we found elevated CRY1 levels in both liver nuclear and whole extracts of *Jmjd5*-null livers compared to wild-type controls (Fig 3D and 3E and S6 Fig). In nuclear extracts from JMJD5-null fibroblasts, CRY1 was slightly increased, even though *Cry1* mRNA levels were much decreased (Fig 3F), which is the exact same situation observed in livers of FBXL3 mutant animals [9].

### JMJD5 connects the proteasome and CRY1

**Fig 4:**
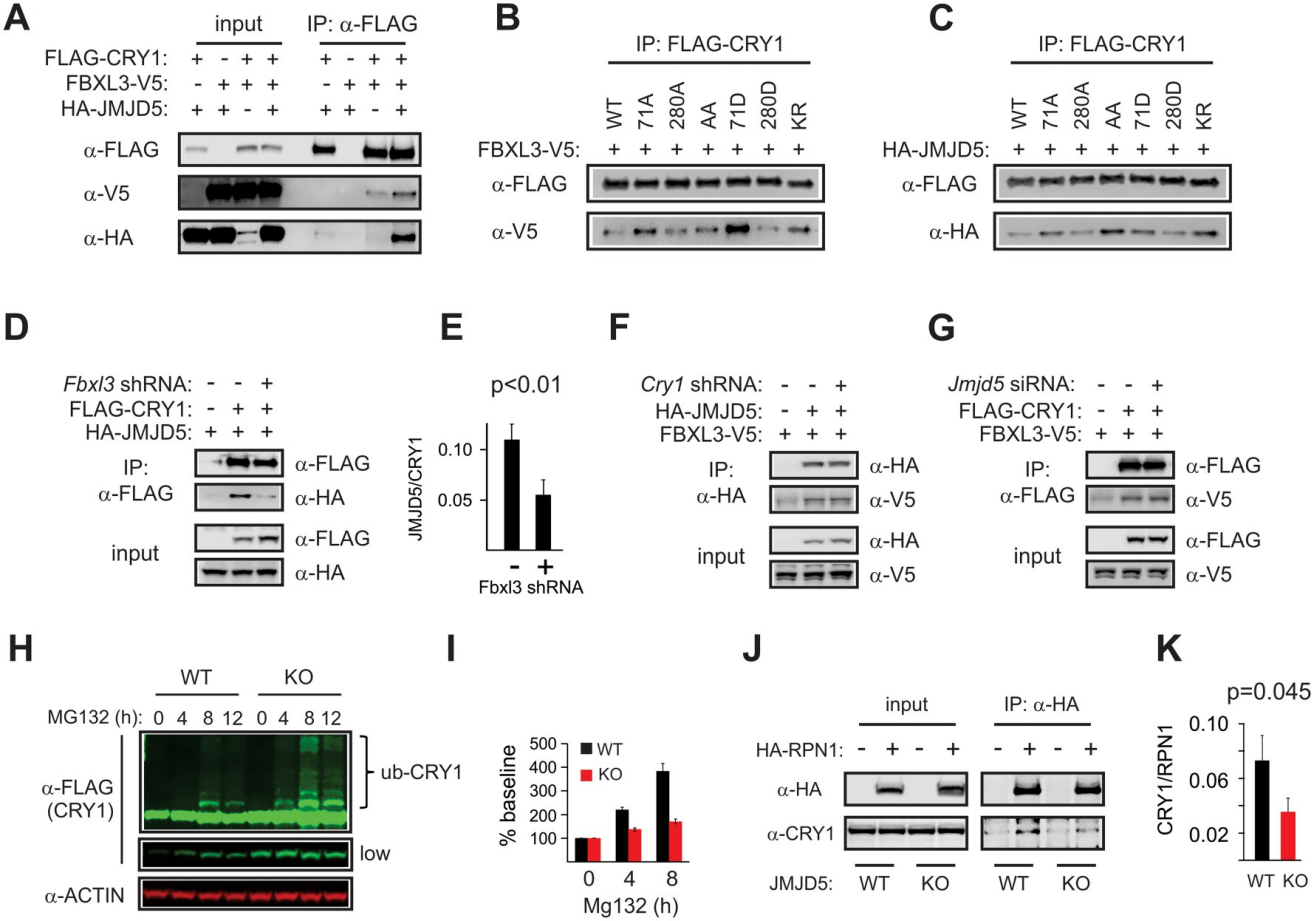
JMJD5 forms a complex with CRY1–FBXL3 and leads to CRY1 degradation. (A) JMJD5 interacts with CRY1. Co-IP experiments show the binding profile of (B) FBXL3 and (C) JMJD5 with CRY1 WT and the indicated CRY1 mutants. Inputs for (B) and (C) are shown in S7 Fig. (D) Knockdown of FBXL3 decreases JMJD5 co-IP with CRY1. Densitometric analysis showing ratio of JMJD5 to CRY1 signals from these experiments (mean ± SEM, *n* = 4) are shown in (E). (F) CRY1 knockdown has no effect on JMJD5–FBXL3 interactions. (G) JMJD5 knockdown does not alter CRY1–FBXL3. (H) CRY1 accumulation in response to MG132 treatment is decreased in JMJD5-null cells compared to controls, whereas ubiquitylation is unaffected (S10 Fig). (I) Quantification of four independent MG132 block experiments shown as baseline-normalized of total CRY1 signal (mean ± SEM, *n* = 4). Regions used for quantification are described in S10 Fig. Twelve-hour time points were omitted as significant cell death was observed. (J) Co-IP of HA-RPN1 and endogenous CRY1 shows diminished binding *Jmjd5−/−* MEFs, quantified in (K), presented as CRY1:RPN1 signal ratios (mean ± SEM, *n* = 6). CRY, CRYPTOCHROME; FBXL3, F-box/leucine-rich repeat protein 3; IP, immunoprecipitation; KO, knockout; JMJD5, JmjC domain–containing protein 5; MEF, mouse embryo fibroblast; RPN1, proteasome regulatory particle non-ATPase 1; shRNA, short hairpin RNA; siRNA, small interfering RNA; WT, wild type.

To determine whether JMJD5 participates directly in regulation of CRY1 degradation, we interrogated its ability to associate with CRY1–FBXL3 complexes. In coimmunoprecipitation studies, we found that JMJD5 interacts with CRY1, but not CRY2, and that this association was enhanced when FBXL3 was coexpressed (Fig 4A and S9 Fig).

CRY1 degradation by FBXL3 is induced via AMPK-mediated phosphorylation of CRY1 residues S71 and S280 [14]. Interaction analyses between FBXL3 and CRY1 constructs harboring phospho-null or phospho-mimetic mutations at these sites showed that although phosphorylation of CRY1 by AMPK increases its binding to FBXL3, it is not required for basal interaction between these two proteins [14]. In a series of coimmunoprecipitation experiments, we found that the binding pattern of JMJD5 to the different phosphosite mutants tested by Lamia and colleagues [14] and to a non-ubiquitylatable CRY1 mutant [13] paralleled that of FBXL3, which further confirmed the existence of CRY1–FBXL3–JMJD5 complexes (Fig 4B and 4C). Association of JMJD5 with CRY1 is dependent on the presence of FBXL3, as RNA interference (RNAi)-mediated knockdown of the latter led to a decrease of CRY1–JMJD5 association (Fig 4D and 4E). In contrast, knockdown of CRY1 did not impact FBXL3 association with JMDJ5, nor did knockdown of JMJD5 abrogate CRY1–FBXL3 interactions (Fig 4F and 4G). Together, these data suggest that FBXL3 bridges the interaction between CRY1 and JMJD5.

As FBXL3 mediates CRY1 ubiquitylation, we assessed whether a defect in this process was responsible for the increased CRY1 levels we observed in a JMJD5-null genetic background. To achieve this, we treated control and JMJD5-null MEFs that expressed FLAG-tagged CRY1 with MG132 to block proteasomal degradation (Fig 4H). Ubiquitylation was not affected. At any given timepoint, the intensity of ubiquitylated CRY1 signal was greater in JMJD5-null than in control cells. However, non-ubiquitylated CRY1 was also increased so that the ratio of these two remains unaffected (S10C Fig), indicating that ubiquitylation of CRY1 was normal. We noted that total CRY1 levels in *Jmjd5+/+* MEFs increased to 400% of baseline levels after 8 hours of MG132 treatment, yet no similar increase over baseline occurred in *Jmjd5−/−* cells (Fig 4I and S10 Fig). Quantification of the non-ubiquitylated band alone yielded similar results (S10 Fig).

These results suggest a reduction in CRY1 degradation by the proteasome in JMJD5-null cells, even while the normal process of ubiquitylation is unaffected. Coincidentally, JMJD5 has been reported to copurify with 19S proteasome regulatory particle non-ATPase 1 (RPN1), the largest 19S proteasome cap subunit [33]. Of note, RPN1 constitutes a docking site for shuttling proteins that help target ubiquitylated substrates to the proteasome [34]. Based on these observations, we hypothesized that JMJD5 was required for normal CRY1 interaction with the proteasome. To test this, we transfected *Jmjd5+/+* and *Jmjd5−/−* cells with an HA-RPN1 expression construct and assessed its ability to associate with endogenous CRY1. We found that CRY1 association with RPN1 was significantly diminished in JMJD5-null cells (Fig 4J and 4K), which argues that JMJD5 facilitates CRY1 targeting to the proteasome.

### CRY1 stability influences its function

**Fig 5:**
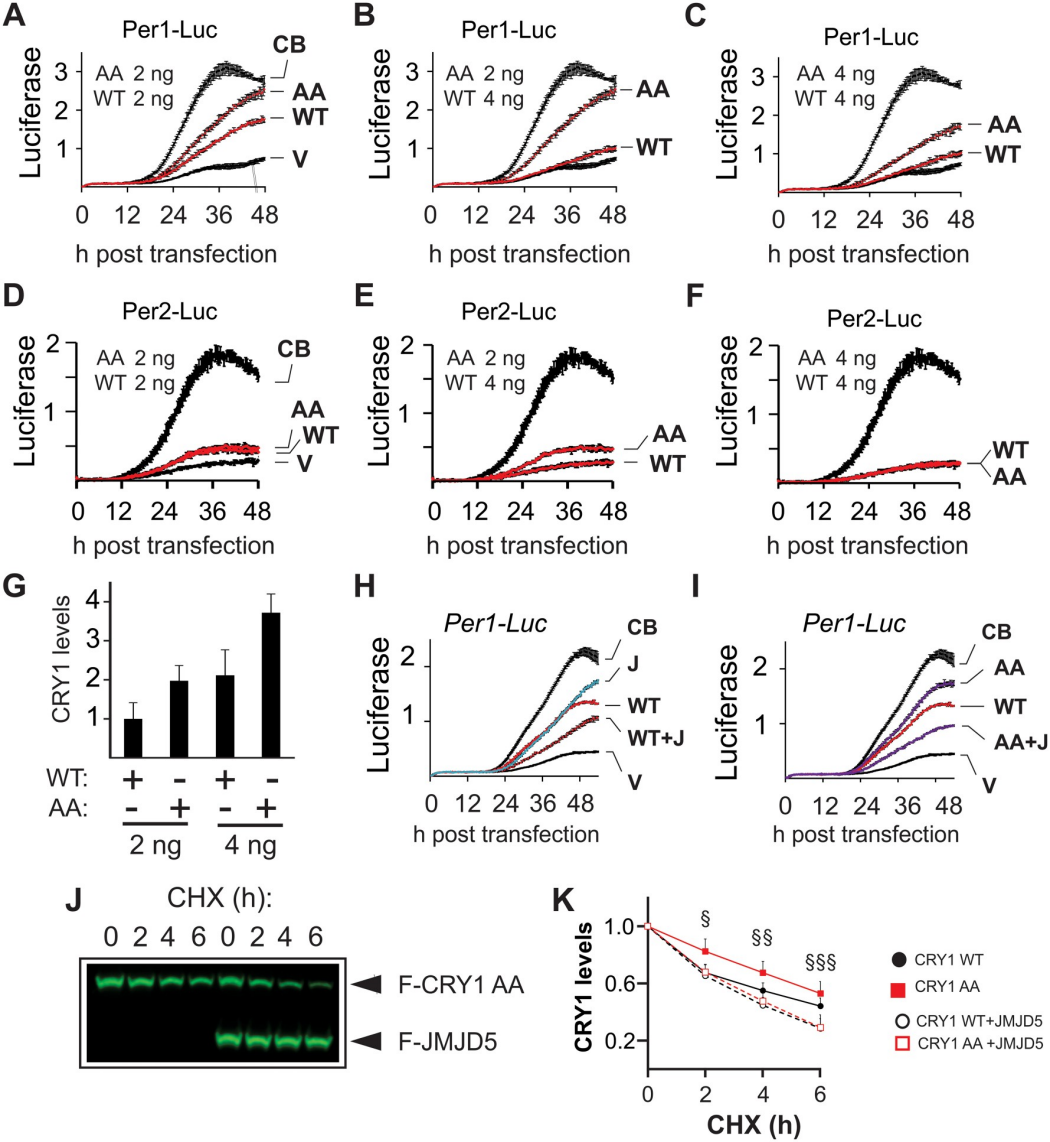
Relationship between CRY1 stability and function. (A–F) Real-time luciferase measurements of *Per1* and *Per2* promoter activity in non-oscillating HEK293T cells show repression of CB by WT CRY1 or AA (mean ± SD, counts ×10^3^). (G) CRY1 levels observed in transfections with the indicated plasmid amounts (mean ± SD, *n* = 3). (H) JMJD5 (denoted by a “J”) cooperates with WT CRY1 to repress CB and (I) compensates for the decreased repressive activity of AA. (J) JMJD5 destabilizes FLAG-AA in a CHX chase assay in HEK293T cells. (K) Quantification of comparing effect of JMD5 on CRY1 AA (J) versus WT CRY1 (data from Fig 3A). §, AA versus AA+JMJD5 *p* < 0.02. §§, WT versus WT+JMJD5 *p* < 0.01; AA versus AA+JMJD5 *p* < 0.01. §§§, WT versus WT+JMJD5 *p* < 0.01; AA versus AA+JMJD5 *p* = 0.05 (WT *n* = 8, others *n* = 5, mean ± SEM, one-tailed Mann-Whitney U-test). AA, CRY1^71A/280A^ mutant; CB, circadian locomotor output cycles protein kaput–brain and muscle ARNT-like protein 1; CHX, cycloheximide; CRY, CRYPTOCHROME; HEK293T, human embryonic kidney 293T; JMJD5, JmjC domain–containing protein 5; V, vector; WT, wild-type.

The seeming paradox posed by the repressive effect of JMJD5 on CLOCK–BMAL1 while simultaneously promoting CRY1 degradation could be resolved if the repressive function of CRY1 was coupled to its degradation. To test this possibility, we performed real-time luciferase assays in non-oscillating HEK293T cells to compare the repressive potential of wild-type to the stable CRY1^71A/280A^ mutant. Consistent with the idea that the repressive function of CRY1 is linked with its degradation, CRY1^71A/280A^ repression of CLOCK–BMAL1 activation of a *Per1*-luciferase reporter was markedly impaired, a defect that was most pronounced when comparing conditions with similar protein levels of wild-type and mutant CRY1 (Fig 5A–5C and 5G).

Repression of CLOCK–BMAL1 by CRY1^71A/280A^ was much less impacted on a *Per2*-luciferase reporter construct (Fig 5D–5F). As JMJD5 inhibition of CLOCK–BMAL1 was lower on a *Per2* than on a *Per1*-luciferase construct (Fig 2A and 2B, S4B and S4C Fig), it is possible that these observations reflect differences in the regulation of these promoters, consistent with previous reports of differential regulation of PER genes [35–37].

Next, we determined whether JMJD5 could act in concert with CRY1 to repress CLOCK–BMAL1. To this end, we measured the repressive activity of wild-type CRY1 in the presence or absence of JMJD5 coexpression (Fig 5H and 5I). To be able to determine the existence of either cooperation or synergism between CRY1 and JMJD5, we transfected suboptimal amounts of CRY1 and JMJD5 plasmids and found that they co-repressed CLOCK–BMAL1. We also observed that the stable CRY1^71A/280A^ mutant cooperated with JMJD5 to an extent similar to the wild type, indicating that JMJD5 could rescue CRY1 activity (Fig 5H and 5I, S11 Fig). Consistently, JMJD5 coexpression destabilized CRY1^71A/280A^ to the same extent as wild-type CRY1 (Fig 5J and 5K). A possible explanation for the ability of JMJD5 to rescue CRY1^71A/280A^ is that increased JMJD5 availability in the context of basal FBXL3–CRY1 interaction leads to increased proteasome degradation. In all, these results strongly support the idea that the repressive ability of CRY1, at least in some contexts, is linked to its degradation.

### JMJD5 is required for AMPK–FBXL3-induced CRY1 degradation

**Fig 6:**
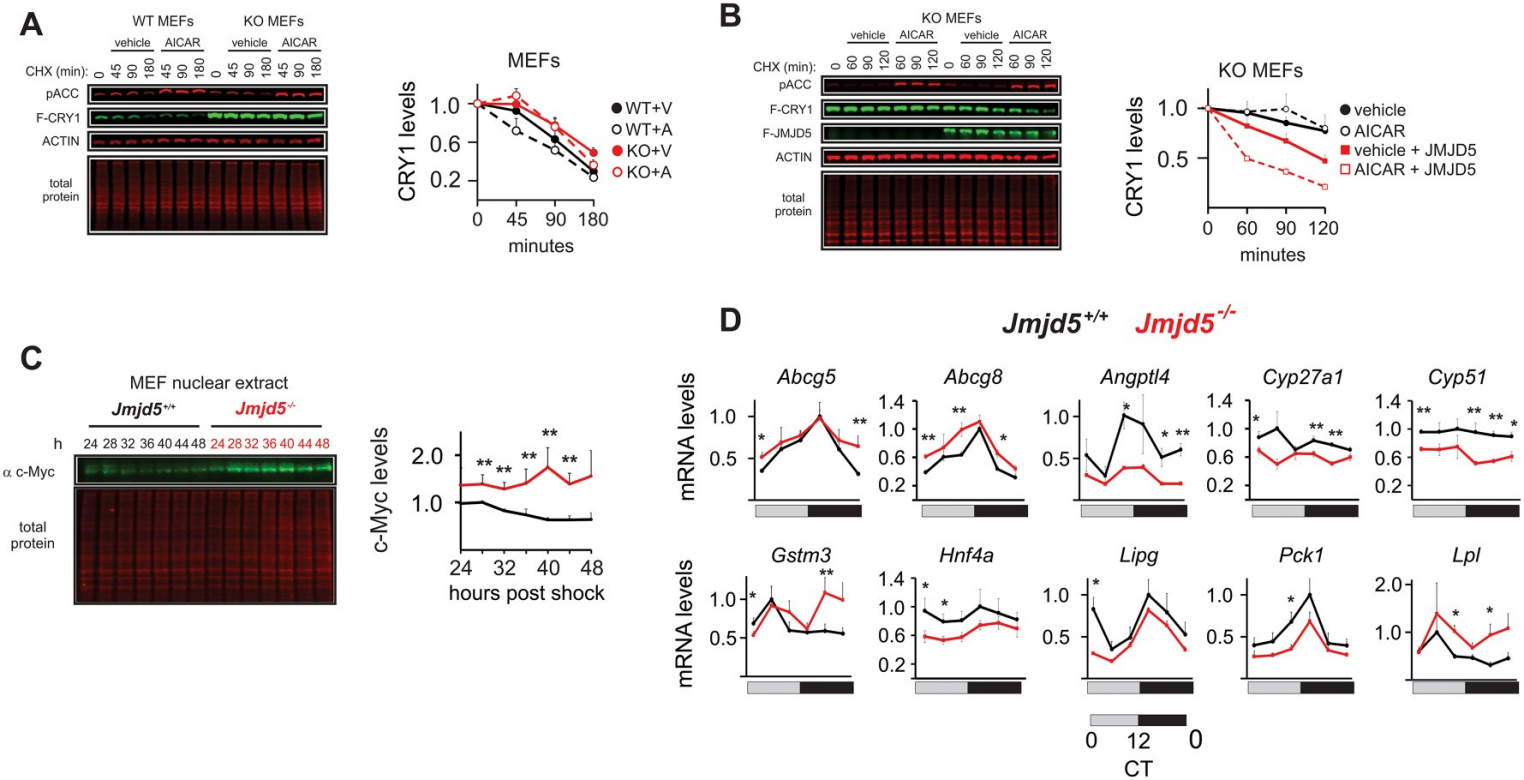
JMJD5 ablation leads to disruption in physiological functions of CRY1. (A) AICAR fails to induce CRY1 degradation in a JMJD5-deficient cellular background (WT *n* = 3, KO *n* = 6, mean ± SEM). (B) Reconstitution of JMJD5 into *Jmjd5*^*−/−*^ rescues CRY1 degradation induced by AMPK activation (WT *n* = 4). (C) Endogenous c-Myc protein levels were measured in circadian timeline from *Jmjd5*^*+/+*^ and *Jmjd5*^*−/−*^ MEFs (mean ± SEM, *n* = 3, one-tailed permutation test **p < 0.01). (D) JMJD5-deficient livers show dysregulation in the mRNA levels of genes regulated by NHR partners of CRY1 (mean ± SEM, *n* = 4; one-tailed permutation test, *p < 0.05, **p < 0.005). AICAR, 5-Aminoimidazole-4-carboxamide 1-β-D-ribofuranoside; AMPK, AMP-regulated protein kinase; CHX, cycloheximide; CRY1, CRYPTOCHROME 1; CT, circadian time; JMJD5, JmjC domain–containing protein 5; KO, knockout; MEF, mouse embryo fibroblast; NHR, nuclear hormone receptor; WT, wild type.

A key feature of the circadian oscillator is its ability to integrate environmental and cellular information with its machinery. This occurs via modulation of its different molecular components by different signaling pathways. AMPK is a master regulator of energy homeostasis that relays information to the circadian clock via CRY1 [14]. As JMJD5 is required for normal CRY1 degradation, we next explored whether it played a role in AMPK-induced CRY1 degradation. To do this, we assessed the effect of AMPK activation on CRY1 levels in wild-type and *Jmjd5−/−* MEFs [28]. In the absence of JMJD5, the basal stability of CRY1 was much greater than in wild-type cells, and AMPK activation by 5-Aminoimidazole-4-carboxamide 1-β-D-ribofuranoside (AICAR) treatment failed to induce the rapid destabilization of CRY1 seen previously by Lamia and colleagues (Fig 6A) [14]. In contrast, reconstitution of JMJD5 sensitized CRY1 levels to the effects of AMPK activation (Fig 6B). Together, these data indicate that JMJD5 has a critical role in control of CRY1 stability by AMPK–FBXL3 axis.

### JMDJ5 deficiency broadly impacts CRY1 and FBXL3 functions

We next asked whether JMJD5 impinges on other biological functions of FBXL3 or CRY1 besides circadian transcription. We first looked at c-MYC levels because its stability is regulated by FBXL3 in conjunction with CRY2 [38]. We interrogated nuclear extracts of control and *Jmjd5*^*−/−*^ MEFs. Across all time points assessed, we found elevation of c-MYC levels in the absence of JMJD5 (Fig 6C).

Next, since CRY1 interacts with and modulates the activity of several NHRs [39,40], we assessed whether hepatic JMJD5 ablation impacted the expression profile of genes regulated by NHR partners of CRY1 (Fig 6D). In JMJD5-null livers, we observed increased levels of genes regulated by liver X receptor α and β (LXRα/β) and liver receptor homolog 1 (LRH1) (*Abcg5* and *Abcg8)* [41–43], peroxisome proliferator–activated receptor δ (PPARδ) (*Lpl*) [44], and pregnane X receptor (PXR) (*Gstm3*) [45,46]. On the other hand, in the absence of JMJD5, we saw decreases in expression of genes regulated by hepatocyte nuclear factor 4α (HNF4α) and PPARγ (*Cyp27a1)* [47,48] and by glucocorticoid receptor (GR; *Angptl4*, *Pck1*, *Lipg*) [39,49,50]. In contrast to increased *Abcg5* and *Abcg8* levels, the expression of another LXR target, *Cyp51* [51], was decreased in *Jmjd5*^*LKO*^ livers. Finally, we also found decreased expression of HNF4α, an NHR that regulates a broad range of hepatic processes and whose promoter region is bound by CRY1 [52].

## Discussion

In this study, we shed light on the mechanisms by which JMJD5 participates in the circadian oscillator. Specifically, we show that JMJD5 plays a role in CRY1 function and in the regulation of stability.

First, JMJD5 expression destabilizes CRY1 but not other circadian proteins. Our data indicate that FBXL3–JMJD5 complexes promote CRY1 degradation by the proteasome. In agreement with this, CRY1–proteasome association is greatly diminished in the absence of JMJD5. We found that although JMJD5 is required for normal CRY1 degradation, it nonetheless cooperates with CRY1 to repress CLOCK–BMAL1, which indicates that CRY1 destabilization and function are, in some cases, positively linked. Indeed, repression of CLOCK–BMAL1 by a degradation-resistant CRY1 mutant is drastically impaired, and JMJD5 simultaneously rescues its functional and stability defects. Though the phenomenon of activation-coupled degradation has been observed in other tightly controlled transcription factors—including the Aryl hydrocarbon Receptor (AhR), Estrogen Receptor alpha (ERα), Sma and Mad homolog 2 (SMAD2), signal transducer and activator of transcription (STATS), and even CLOCK–-BMAL1 [53,54]—this is the first time it has been described for a circadian repressor. Until now, the view regarding the relationship between the repressive function of CRY1 and its protein levels has been that these have direct correlation. As this perspective has been largely shaped by studies involving mechanisms that regulate levels of both CRY1 and CRY2, it is likely that, in such context, differences in the function and regulation between the two CRY paralogs may be obscured. Since the mechanism we describe here is specific to CRY1, we are now able to better define how regulation of CRY1 levels relates to its activity.

Cells deficient in JMJD5 exhibit dysregulation in circadian gene expression, albeit with a pattern diverging from simple E-box regulation, which is consistent with previous studies. A Gene Dosage Network Analysis (GDNA) by Baggs and colleagues, for instance, showed that clock gene expression responses to circadian network perturbations are complex, depend on the specific oscillator component that is being disrupted, and do not always follow predicted changes based on transcriptional relationships [55]. In that study, siRNA-mediated depletion of *Clock* reduces *Nr1d1* and *Nr1d2* levels, has a marginal impact on *Per1*, has no effect on *Cry2* and *Per2*, and results in slight increases in *Cry1* mRNA, all of which are canonical target genes of CLOCK–BMAL1-mediated activation. In addition, *Cry1* depletion in U2OS cells clearly increases levels of *Per2* and *Cry2* but has no apparent impact on *Per1*, *Nr1d1*, or *Nr1d2*. However, our observations in both *Jmjd5*-deficient cells and liver do have overlap with findings by Baggs and colleagues. Specifically, the opposite changes in *Per1* and *Per2* mRNA expression we observed in JMJD5-null cells (increase and decrease, respectively) are consistent with the unidirectional *Period* paralog compensation in gene expression observed by Baggs and colleagues, in which *Per1* depletion increases *Per2* levels but not in the reverse [55]. Our functional assays suggest that, at least in certain contexts, JMJD5 may have a more prominent role in control of *Per1* transcription than in that of *Per2* (Fig 2B and S4 Fig). Consistently, increased CRY1 stability had a much greater impact to repress a *Per1*-luciferase construct than one driven by the *Per2* promoter (Fig 5A–5F). These observations may reflect reports that control of *Per1* and *Per2* transcription is not identical and raises the intriguing possibility that JMJD5 has a role in the mechanisms behind paralog compensation.

Similarly, complex patterns of clock expression occur in tissues of clock component knockout mice. Single knockout of CRY1 or single knockout of CRY2 affects transcription not only differently across genotypes but also across tissues within a genotype [56]. For example, *Per2* mRNA levels in the liver of CRY1 knockout mice are elevated and rhythmic but are arrhythmic and mostly reduced in the cerebellum. CRY2 ablation, on the other hand, does not result in derepression of *Per2* in liver, a canonical E-box driven target, but quite the opposite. In CLOCK knockout mice, *Per1* mRNA levels drop in the hypothalamic suprachiasmatic nucleus (SCN) but rise in the liver, whereas the phase of *Per2* mRNA rhythm is shifted without any impact on its levels [57]. In contrast, *Per2* mRNA in CLOCK-null mouse liver is elevated only during the nadir of expression, whereas Dbp and *Nr1d1* levels are decreased despite drastically elevated *Bmal1* gene expression [57]. Finally, a recent study by Ramanathan and colleagues found that knockdown of canonical clock genes (e.g., *Cry1*, *Per1*, *Per2*, *Nr1d1*) do not always result in the same circadian effect in different cell lines [58]. Altogether, these observations indicate that deletion of a single clock regulator—even of canonical clock components—can lead to nonintuitive effects, which help explain our observations here.

Hepatic ablation of JMJD5 also resulted in abnormalities in circadian gene expression. As with *Jmjd5−/−* cells, we observed elevation of *Per1* levels with a simultaneous decrease in *Per2* levels, again suggestive of paralog compensation. As with *Per* genes, paralog compensation in *Cryptochromes* occurs unidirectionally, so that knockdown of *Cry1* gene expression in U2OS cells leads to an increase in *Cry2* transcript but not vice versa. In JMJD5-deficient liver, we noted a slight increase in *Cry2* mRNA levels, which could reflect a decrease in CRY1 function even if *Cry1* transcripts remain unaltered. In contrast to cells, we observed no changes in the levels of *Bmal1*, *Dbp*, *Cry1*, or *Nr1d1* transcripts in JMJD5-null liver tissue. A possible explanation for the differences in the impact that JMJD5 deletion has on cells and liver is that circadian clock control is not identical in all cell types and/or due to divergence in how the clock is regulated (e.g., differences in paralog levels) in cultured cells versus in vivo. Knockdown of canonical clock genes (e.g., *Cry1*, *Per1*, *Per2*, *Nr1d1*) does not always result in the same circadian effect in different cell lines [58], which lends further support to this idea.

In a previous study, Huber and colleagues demonstrated that CRY2–FBXL3 specifically regulates c-MYC protein stability. Likewise, we find that c-MYC levels are affected, suggesting that JMJD5 may impact FBXL3 function beyond CRY1 degradation, yet this effect could be indirect, given that JMJD5 did not impact or interact with CRY2. Huber and colleagues also noted a moderate yet noticeable increase in overexpressed c-MYC upon *Cry1* ablation [38]. Thus, elevation of c-MYC levels in the absence of JMJD5 is consistent with a deficit in CRY1 function. A possibility is that ablation of JMJD5 disturbs the balance between CRY1–FBXL3 and CRY2–FBXL3 complexes. Nonetheless, the precise mechanisms by which JMJD5 influences c-MYC function remain to be discovered.

Second, CRY1 interacts with and participates in NHR-mediated transcription control. In JMJD5-deficient livers, we found abnormal expression of several genes regulated by one or more NHRs that are known to interact strongly with CRY1. The genes we assessed are known to code for important components of metabolic processes, including cholesterol metabolism (*Abcg5*, *Abcg8*, *Cyp27a1*, *Cyp51*), lipid metabolism (*Angptl4*, *Lpl*, *Lipg*), glucose metabolism (*Pck1)*, and xenobiotic detoxification (*Gstm3*); their dysregulation suggests that JMJD5 function may have an important role in regulation of liver physiology by CRY1. We found both up-regulation and down-regulation in the expression of these genes, which is reminiscent of what we observed in oscillator components. We found decreased expression of genes regulated by NHRs that are repressed to a similar extent by both CRY1 and CRY2 (e.g., the GR target *Angptl4*), which is consistent with increased CRY1 levels. In contrast, genes controlled by PPARδ and PXR, which are more strongly repressed by CRY2 than by CRY1, were moderately derepressed [40]; a possible explanation is that this effect is due to a decreased association of CRY2 with PPARδ and PXR as a consequence of increased CRY1 availability.

Finally, our observation that JMJD5 seems to impinge on the core clock through CRY1 but not through other core components is intriguing. Several observations suggest that CRY1 serves a unique repressive function. First, CRY1 is able to bind and repress CLOCK–BMAL1 independently of PER [59]. In liver, CRY1 has a markedly different temporal genomic occupancy pattern than that of other circadian oscillators [52]. Furthermore, a recent study found that under certain conditions, most circadian proteins are only detectable as part of a large multiprotein complex, with the exception of CRY1 and CKIδ; in that study, both were detected as uncomplexed from other clock components [60]. When considered together, these observations support the existence of CRY1-specific regulatory mechanisms and thereby suggest that CRY1 constitutes a unique node through which the molecular oscillator machinery is fine-tuned.

## Materials and methods

### Ethics statement

This work involved the killing of animals by cervical dislocation, as approved by the University of Kansas Medical Center Institutional Animal Care and Use Committee (IACUC) (protocol # 2015–2292).

### Cell culture and transfections

HEK293T cells were purchased from the American Type Culture Collection (ATCC). Cells were cultured in Dulbecco’s Modified Eagle Medium (DMEM) (Corning Cat# 10-013-CV) supplemented with 10% FBS (Atlanta Biologicals Cat# S11595H) and 1% antibiotics and antimycotics (Thermo Fisher Cat# 15240062) in a 37 °C incubator maintained at 5% CO_2_. Transfections were performed using Trans-IT LT1 (TLT-1) (Mirus Bio Cat# MIR 2304) according to the manufacturer’s instructions (specific conditions described below).

### Cycloheximide chase experiments

HEK293T cells were seeded out in 24-well plates at 80,000 cells per well. Twenty-four hours later, they were transfected with 200 ng of FLAG-CRY1, 40 ng of FLAG-JMJD5, 260 ng of pCDNA3.1 vector (500 ng total), and 1.5 μl of transfection reagent. Forty-eight hours post transfection, cycloheximide (Sigma Cat# C7698-1G) was added to each well to a final concentration of 100 μg/ml, and the cells were harvested every 2 hours.

*Jmjd5+/+* and *Jmjd5−/−* immortalized MEFs were obtained from Dr. Ralf Janknecht’s laboratory and have been previously described [28]. *Jmjd5+/+* and *Jmjd5−/−* MEFs were seeded out in 6-well plates at a concentration of 350,000 cells per well. After 20 hours, the cells were transfected with 1 μg of FLAG-CRY1 and 1.5 μl of TLT-1. Forty-eight hours post transfection, the cells were treated with a mixture of cycloheximide (100 μg/ml) ± 3 mM AICAR. For rescue experiments, *Jmjd5−/−* were seeded out in 6-well plates at a concentration of 350,000 cells per well. After 20 hours, the cells were transfected with 1 μg of FLAG-CRY1 and 100 ng of FLAG-JMJD5 (or pCDNA 3.1+ vector). Forty-eight hours post transfection, the cells were treated with a mixture of cycloheximide (100 μg/ml) ± 3 mM AICAR and harvested at the indicated time points.

*Jmjd5+/+* and *Jmjd5−/−* MEFs were seeded out in 6-well plates at 350,000 cells per well and 24 hours later were transfected with 1 μg FLAG-CRY1. After 48 hours of transfection, the cells were treated with 10 μM MG132, and cells were harvested every 4 hours for 12 hours.

### Plasmids

FLAG-CRY1, FLAG-CRY1S71A, FLAG-CRY1S280A, and FLAG-CRY1AA in pcDNA3.1+ expression backbone were a gift from Katja Lamia. FLAG-JMJD5 and V5-JMJD5 in the pEV3S backbone and HA-JMJD5 in the pQCXIH backbone were generated by Ralf Janknecht. The CRY1(K:R)-HA construct was a kind gift of Dr. Joe Takahashi.

### Luciferase assays

Real-time luciferase assays were performed in a 96-well plate format by reverse transfecting HEK293T (40,000 cells per well) with a total of 250 ng of DNA (10 ng of pGL3 *Per1*: *Luc* reporter, 30 ng CMV-CLOCK, 10 ng of CMV-*Bmal1*, and up to 200 ng of test plasmids) and 7.5 μl of TLT-1. The cells were seeded out in phenol red–free DMEM/Ham’s F-12 50/50 mix (Corning Cat# 16-405-CV) supplemented with 10% FBS, 1% Antibiotic-Antimycotic (Life Technologies), 25 mM HEPES, and 125 μM of D-Luciferin. The plate was sealed tight with TempPlate Optical film (USA Scientific). The plate was immediately transferred to the Tecan Infinite M200 maintained at 37 °C, and luminescence was measured in kinetic mode (every 20 minutes) for at least 72 hours. To determine the relative expression of flag components, lysates were prepared at the time corresponding to the peak of CLOCK–*Bmal1* activity and analyzed by western blots.

### Real-time circadian bioluminescence

*Jmjd5+/+* and *Jmjd5−/−* MEFs were seeded out in a 35-mm dish with 350,000 cells per dish. Sixteen hours later, they were transfected with 2 μg of a *Per2-Luc* reporter construct. Forty-eight hours post transfection, they were shocked with 0.1 μM dexamethasone for 2 hours. The media were replaced with DMEM:F12 media without phenol red containing 1% antibiotic-antimycotic, 10% FBS, and 25 mM HEPES. The plates were sealed tight and placed in an incubating luminometer (Atto Kronos), and the luminescence was measured for 5 days.

### Preparation of protein lysates, immunoprecipitations, and immunoblotting

Whole-cell lysates from cells and livers were prepared using lysis buffer containing 150 mM NaCl, 50 mM Tris-HCl, 0.5% TX-100, 0.5% NP-40, 0.25% Sodium Deoxycholate 0.025% SDS along with EDTA-free protease inhibitor cocktail and phosphatase inhibitors (Roche Cat# 4693159001 and 4906845001, respectively). Briefly, cells or crushed tissue was incubated with lysis buffer for 30 minutes on ice and then spun at 10,000 rpm for 10 minutes at 4 °C. To prepare the nuclear lysates, liver tissue was homogenized in a hypotonic buffer (10 mM Tris HCl [pH 8.0], 10 mM KCl, 0.5 mM MgCl_2_) using a Dounce homogenizer. The homogenate was then centrifuged at 800*g* for 5 minutes at 4 °C. The pellet was resuspended in S1 buffer (0.25 M sucrose and 10 mM MgCl_2_), layered onto a sucrose cushion S2 (0.88 M sucrose and 0.5 mM MgCl_2_), and centrifuged at 3,000 rpm for 10 minutes at 4 °C. The supernatant was carefully discarded, and the pellet was resuspended in buffer B2 (10 mM Tris HCl [pH 8.0] and 300 mM NaCl) and incubated on ice for 45–60 minutes. This was then centrifuged at 3,000 rpm for 10 minutes at 4 °C. The resulting supernatant was the nuclear extract and was used in subsequent applications. Nuclear extracts from the MEFs were prepared using this protocol, and confluent 35-mm dishes of cells were used.

For immunoprecipitations using M2 FLAG magnetic beads (Sigma Cat# M8823-1ML), cells were lysed using a lysis buffer containing 200 mM NaCl, 50 mM Tris-HCl, 1% TX-100, and 1% NP-40 supplemented with phosphatase and protease inhibitors. HEK293T cells were seeded out in 6-well plates and transfected the next day with a total of 2.5 μg of DNA (1.5 μg V5-FBXL3, 400 ng FLAG-CRY1, and 600 ng of HA-JMJD5). Forty-eight hours post transfection, the cells were lysed, incubated on ice for 30–45 minutes, and spun at 10,000 rpm for 10 minutes at 4 °C. The supernatant was incubated with the M2 beads overnight at 4 °C while tumbling. Subsequently, the beads were washed 3 times with chilled 1X TBS for 5 minutes each. The protein was eluted from the beads with equal volume of 3X flag peptide (Sigma Cat# F4799-4MG). The eluate was boiled in NuPAGE LDS Sample Buffer and reducing buffer and subjected to SDS-PAGE-immunoblot analysis.

HEK293T cells were transfected with 1.5 μg V5-FBXL3, 400 ng HA-CRY1K:R, and 600 ng of V5-JMJD5. Forty-eight hours post transfection, the cells were lysed using the lysis buffer described above and immunoprecipitated with HA antibody bound to protein G beads for 1 hour at 4 °C. The beads were washed 3 times with chilled 1X TBS for 5 minutes with mild tumbling. The bound proteins were eluted by boiling in sample buffer and subjected to SDS-PAGE-immunoblot analysis.

*Jmjd5+/+* and *Jmjd5−/−* MEFs were transfected with HA-RPN1 and 48 hours later were lysed with buffer containing 400 mM NaCl, 50 mM Tris-HCl, 1% TX-100, and 0.25% Sodium Deoxycholate, supplemented with phosphatase and protease inhibitors. The lysates were incubated on ice for 30–45 minutes and spun at 10,000 rpm for 10 minutes at 4 °C. Protein G beads were prebound with anti-HA tag antibody. The lysates were incubated with the antibody–bead complexes for 1 hour at 4 °C and washed 5 times with 1X TBS containing 0.5% Triton X-100. The bound proteins were eluted by boiling in sample buffer and subjected to SDS-PAGE-immunoblot analysis to detect endogenous CRY1 levels bound to RPN1.

FLAG M2 (Sigma), Anti-HA (12CA5), V5 (Abcam), and Anti-CRY1 were the antibodies used in this study. Anti-CRY1 (687) antibody was a kind gift from Satchin Panda.

### RNA preparation and qPCR analysis

*Jmjd5+/+* and *Jmjd5−/−* MEFs were seeded out in 6-well plates at 350,000 cells per well and shocked 48 hours later with 100 nM of Dexamethasone for 2 hours before supplementing with fresh medium. Thirty-six hours post shock, the cells were harvested using 1 ml TRIzol (Fisher Scientific Cat# 15596026) and stored at −80 °C every 4 hours. Total RNA was then prepared using the manufacturer’s instructions. For real-time qPCR, 1 μg of RNA was reverse transcribed to cDNA using qScript cDNA SuperMix (Quanta Biosciences Cat# 95048–025). FastStart Universal SYBR Green Master (Rox) (Roche Cat# 4913850001) was used to perform the qPCR reaction in a BIO-RAD CFX384 Touch Real-Time PCR System.

### qPCR primers

mDbp-F: GAG CCT TCT GCA GGG AAA CA
mDbp-R: GCC TTG CGC TCC TTT TCC
mCLOCK-F: AGAACTTGGCATTGAAGAGTCTC
mCLOCK-R: GTCAGACCCAGAATCTTGGCT
m*Bmal1*-F: GCC CCA CCG ACC TAC TCT
m*Bmal1*-R: TGT CTG TGT CCA TAC TTT CTT GG
m*Cry1*-F: ATC GTG CGC ATT TCA CAT AC
m*Cry1*-R: TCC GCC ATT GAG TTC TAT GAT
m*Cry2*-F: GCA GAG CCT GGT TCA AGC
m*Cry2*-R: GCC ACT GGA TAG TGC TCT GG
m*Per1*-F: GCT TCG TGG ACT TGA CAC CT
m*Per1*-R: TGC TTT AGA TCG GCA GTG GT
m*Per2*-F: TCC GAG TAT ATC GTG AAG AAC G
m*Per2*-R: CAG GAT CTT CCC AGA AAC CA
m*Nr1d1*-F: GGA GCT GGG CCT ATT CAC CGC
m*Nr1d1*-R: GCT GCT CCA CCG AAG CGG AA.
m*Jmjd5*-F: CGCAGTCCTCCAGACACACC
m*Jmjd5*-R: CAAGATCACAGGCCTCCCAG
mMrpl46-F: GGTCCGGTCATTTTTTTTGTCA
mMrpl46-R: GGGAGCAGGCATTCCTACAG
mRORA-F: GTGGAGACAAATCGTCAGGAAT
mRORA-R: TGGTCCGATCAATCAAACAGTTC
mABCG5-F: AGG GCC TCA CAT CAA CAG AG
mABCG5-R: GCT GAC GCT GTA GGA CAC AT
mGSTM3-F: CCC CAA CTT TGA CCG AAG C
mGSTM3-R: GGT GTC CAT AAC TTG GTT CTC CA
mLPL-F: GGG AGT TTG GCT CCA GAG TTT
mLPL-R: TGT GTC TTC AGG GGT CCT TAG
mLIPG-F: ATG CGA AAC ACG GTT TTC CTG
mLIPG -R: GTA GCT GGT ACT CCA GTG GG
mPCK1-F: CTG CAT AAC GGT CTG GAC TTC
mPCK1-R: CAG CAA CTG CCC GTA CTC C
mANGPTL4-F: CAT CCT GGG ACG AGA TGA ACT
mANGPTL4-R: TGA CAA GCG TTA CCA CAG GC
hJMJD5-F: GGC CCG TGA TCC TGA AAG G
hJMJD5-R: GGC TCA TTC ACG ATG TAT TTG C
hCRY1-F: ACA GGT GGC GAT TTT TGC TTC
hCRY1-R: TCC AAA GGG CTC AGA ATC ATA CT
hFBXL3-F: GCA GCT TGT GAT ATA CTA TCG CA
hFBXL3-R: TGG TCG AGC AGT TGA AAT AAG TC
hHPRT1-F: CCT GGC GTC GTG ATT AGT GAT
hHPRT1-R: AGA CGT TCA GTC CTG TCC ATA A

### shRNA and siRNA knockdowns

All the shRNA and siRNA knockdowns were performed using transient cotransfection of the constructs along with overexpression constructs of FLAG-CRY1, HA-JMJD5, and V5-FBXL3 in HEK293T cells in 6-well plates.

shRNA hFBXL3 Sigma MISSION shRNA TRCN0000369031 (5′-CCGGCTGATCAGTGTCACGGCTTAACTCGAGTTAAGCCGTGACACTGATCAGTTTTTG-3′), shRNA hCRY1 Sigma MISSION shRNA TRCN0000231065 (5′-CCGGGGAACGAGACGCAGCTATTAACTCGAGTTAATAGCTGCGTCTCGTTCCTTTTTG-3′), siRNA hJMJD5 Sigma MISSION siRNA EHU149061, siRNA universal negative control Sigma MISSION siRNA SIC001.

#### Circadian tissue timelines

Control or *Jmjd5*^*LKO*^ mice between 6 and 9 weeks old were entrained in a light tight chamber to a 12-hour light:12-hour dark cycle for 10 days and released into constant darkness for 24 hours. Mice were then killed every 4 hours for a 24-hour period.

#### JMJD5 conditional knockout mice (Jmjd5^LKO^)

C57BL/6NTac-Jmjd5tm1a(EUCOMM)Wtsi frozen embryos were obtained from the European Conditional Mouse Mutagenesis Program (EM:04155), and embryo reconstitution was performed at Charles River. Born heterozygotes were crossed with the FLP deleter strain B6.129S4-Gt(ROSA)26Sortm1(FLP1)Dym/RainJ (Jackson Laboratory 009086) to excise the lacZ-neo cassette. Thereby, conditional Jmjd5flox knockouts were created, which were bred for several generations onto a C57BL/6 background. To generate liver-specific JMJD5-null animals, Jmjd5flox/flox mice were bred with a transgenic mouse line that specifically expresses CRE in the liver at high levels (albumin promoter–driven CRE; C57BL/6J congenic Alb-CRE, Jackson Laboratories stock 003574. LoxP(−)/Cre+ littermates were used as controls.

### Per1 promoters for E-box-related experiments

The following gBLOCKS were ordered from IDT and cloned into pGL3 BASIC (Promega).

Wild-type E-boxes: AGTGCTAGCCATCACCCACTCACCCCTTAACGACACGTGGGCCCTCAATTGCCCTTCTCTCAGGATCTGAAGGGTCAGAGGAAAGGGTTGGATTCTTTATAACAAGGCTGGGGAGAGGCCAGGGAATGTCAGTCTAGGTTTTTCTCTCTCCCACTTCCCTTGGGTAGCAGACATTTCATTCACCCGGCACCAGGACAGGTGTCTTGTTCTGCCAAGCTGGTCAGTTTAGGAAGTAGGTTTCTCTTGAGCACTTCCTGTGGCCCAGGTATCCTCCCTGAAAAGGGGTAGTTTCCCTCCCTCACTTCCCTTTCATTATTGACGGTGTGAGACATCCTGATCGCATTGGCTGACTGAGCGGTGTCTGAGGCCCTTCAGCCCAGCACCAGCACCCAAGTCCACGTGCAGGGATGTGTGTGACACAGCCCTGACCTCAGTGGGGGCCAGTAGCCAATCAGATGCCAGGAAGAGATCCTTAGCCAACCGGGGGCGGGGCCTGCGGCTCTTCGGGCAGAAGGCCAATGAGGGGCAGGGCCTGGCATTATGCAACCCGCCTCCCAGCCTCGCGGAGCTTCTGGGTTGCAAGCTTAGC.

E-boxes mutated: AGTGCTAGCCATCACCCACTCACCCCTTAACGACAgGTcGGCCCTCAATTGCCCTTCTCTCAGGATCTGAAGGGTCAGAGGAAAGGGTTGGATTCTTTATAACAAGGCTGGGGAGAGGCCAGGGAATGTCAGTCTAGGTTTTTCTCTCTCCCACTTCCCTTGGGTAGCAGACATTTCATTCACCCGGCACCAGGACAGGTGTCTTGTTCTGCCAAGCTGGTCAGTTTAGGAAGTAGGTTTCTCTTGAGCACTTCCTGTGGCCCAGGTATCCTCCCTGAAAAGGGGTAGTTTCCCTCCCTCACTTCCCTTTCATTATTGACGGTGTGAGACATCCTGATCGCATTGGCTGACTGAGCGGTGTCTGAGGCCCTTCAGCCCAGCACCAGCACCCAAGTCCAgGTcCAGGGATGTGTGTGACACAGCCCTGACCTCAGTGGGGGCCAGTAGCCAATCAGATGCCAGGAAGAGATCCTTAGCCAACCGGGGGCGGGGCCTGCGGCTCTTCGGGCAGAAGGCCAATGAGGGGCAGGGCCTGGCATTATGCAACCCGCCTCCCAGCCTCGCGGAGCTTCTGGGTTGCAAGCTTAGC.

## Supporting information

S1 Fig JMJD5 mRNA levels in MEFs and murine liver. mRNA levels of JMJD5+/+ and JMJD5−/− MEFs (mean ± SEM *n* = 3) and mice livers (mean ± SEM *n* = 4) were determined by qPCR analysis. JMJD5, JmjC domain–containing protein 5; MEF, mouse embryo fibroblast; qPCR, quantitative PCR.
(TIF)

S2 Fig Ablation of JMJD5 results in MEFs results in reduced period of oscillation. Real-time bioluminescence measurement from overexpressed *Per2-Luc* promoter in *Jmjd5+/+* and *Jmjd5−/−* MEFs (*n* = 3, mean ± SD). JMJD5, JmjC domain–containing protein 5; MEF, mouse embryo fibroblast.
(TIF)

S3 Fig Generation of *Jmjd5*^*LKO*^ mice. Schema of targeting vector and resulting floxed allele. Exons shown as solid boxes; Frt and loxP sites are respectively shown as green and purple triangles. Frt, flippase recognition target; *Jmjd5*^*LKO*^, JmjC domain–containing protein 5 liver knockout; loxP, locus of X-over P1.
(TIF)

S4 Fig JMJD5 is a repressor of CLOCK–BMAL1. (A) Relative protein levels of FLAG-JMJD5 and FLAG-JMJD5^H321A^ (MUT) in the real-time luciferase assay in Fig 2A, 2B, 2E and 2F. (B and C) Data from Fig 2A and 2B was replotted by dividing the luciferase signal obtained with CLOCK+BMAL1+JMJD5 conditions over that obtained with just CLOCK+BMAL1 at the Per1 (solid circles) and Per2 (open circles) promoters. Panel B corresponds to results obtained with 100 ng of JMJD5 plasmid used and panel A to results obtained with 200 ng of JMJD5 plasmid. BMAL1, brain and muscle ARNT-like protein 1; CLOCK, circadian locomotor output cycles protein kaput; JMJD5, JmjC domain–containing protein 5.
(TIF)

S5 Fig Assessment of core oscillator component stability in the presence and absence of FLAG-JMJD5. (A) FLAG-CLOCK and BMAL, (B) FLAG-PER1, and (C) FLAG-PER2. BMAL, brain and muscle ARNT-like protein; CLOCK, circadian locomotor output cycles protein kaput; JMJD5, JmjC domain–containing protein 5; PER, PERIOD.
(TIF)

S6 Fig JMJD5 ^LKO^ livers show increased CRY1 levels in WLEs. Total protein stain was used as loading control. CRY1, CRYPTOCHROME 1; *Jmjd5*^*LKO*^, JmjC domain–containing protein 5 liver knockout; WLE, whole liver extract.
(TIF)

S7 Fig JMJD5 binding to CRY1 follows a similar pattern as that of FBXL3. Shown are the inputs for the coimmunoprecipitations of FLAG-tagged CRY1 and mutant CRY1s with (A) V5-FBXL3 and (B) HA-JMJD5. CRY1, CRYPTOCHROME 1; FBXL3, F-box/leucine-rich repeat protein 3; JMJD5, JmjC domain–containing protein 5.
(TIF)

S8 Fig Knockdown levels of *Fbxl3*, *Cry1*, and *Jmjd5* in HEK293T cells by shRNA/siRNA was measured by qPCR and normalized to HPRT1. HEK293T, human embryonic kidney 293T; HPRT1, hypoxanthine phosphoribosyltransferase 1 gene; qPCR, quantitative PCR; shRNA, short hairpin RNA; siRNA, small interfering RNA.
(TIF)

S9 Fig FLAG-CRY2 is not able to interact with HA-JMJD5. Immunoprecipitation of FLAG-CRY2 in HEK293T cells shows no interaction with HA-JMJD5 both in the absence and presence of V5 FBXL3. FLAG-CRY1 interaction with HA-JMJD5 is shown as a positive control. * denotes an unknown band observed only when FLAG-CRY2 is transfected. CRY, CRYPTOCHROME; FBXL3, F-box/leucine-rich repeat protein 3; HEK293T, human embryonic kidney 293T; JMJD5, JmjC domain–containing protein 5.
(TIF)

S10 Fig MG132 treatment showed no impairment in the ubiquitylation status of CRY1 in *Jmjd5−/−* cells. (A and B) Shown are the areas of the blot used to quantify the accumulation of CRY1. (C) Quantification of the main band (non-ubiquitylated CRY1) reveals a similar pattern of accumulation as quantifying all the forms of CRY1 as shown in Fig 4I and S10B Fig. (D) Ratio of ubiquitylated forms of CRY1 to non-ubiquitylated forms as shown in (A) reveals that there is no impairment in the ubiquitylation status of CRY1. CRY1, CRYPTOCHROME 1.
(TIF)

S11 Fig JMJD5 cooperates with CRY1 and AA to repress the clock. Real-time luciferase assays from Fig 5H and I represented in the same figure for comparison of repression levels. AA, CRY1^71A/280A^; CRY1, CRYPTOCHROME 1; JMJD5, JmjC domain–containing protein 5.
(TIF)

S12 Fig Anti-CRY1 antibody validation in wild-type and *Cry1−/−* MEFs. CRY1, CRYPTOCHROME 1; MEF, mouse embryo fibroblast.
(TIF)

S1 Data Numerical data underlying figures. (XLSX)

## Abbreviations

AhR: Aryl hydrocarbon Receptor
AICAR: 5-Aminoimidazole-4-carboxamide 1-β-D-ribofuranoside
AMPK: AMP-activated protein kinase
*AtJmjd5*: *Arabidopsis thaliana Jmjd5*
CKI: Casein kinase I
CLOCK: circadian locomotor output cycles protein kaput
CRY: CRYPTOCHROME
CT: circadian time
ERα: Estrogen Receptor alpha
FBXL: F-box/leucine-rich repeat protein
GDNA: Gene Dosage Network Analysis
GR: glucocorticoid receptor
GSK3β: glycogen synthase kinase 3β
HECT: homologous to the E6-AP Carboxyl Terminus
HEK293T: human embryonic kidney 293T
HNF4α: hepatocyte nuclear factor 4 α
JMJD5: JmjC domain–containing protein 5
*Jmjd5^LKO^*: *Jmjd5* liver knockout
KDM2A: lysine-specific demethylase 2A LRH1, liver receptor homolog 1
LXRα/β: liver X receptor α and β
MEF: mouse embryo fibroblast
NHR: nuclear hormone receptor
PER: PERIOD
PPAR: peroxisome proliferator–activated receptor
PXR: pregnane X receptor
RING: really interesting new gene
RNAi: RNA interference
RPN1: 19S proteasome regulatory particle non-ATPase 1
SCN: suprachiasmatic nucleus
siRNA: small interfering RNA
SMAD2: Sma and Mad homolog 2
STATS: signal transducer and activator of transcription
