# Correction: JMJD5 links CRY1 function and proteasomal degradation

**DOI:** 10.1371/journal.pbio.3000454

**Published:** 2019-08-21

**Authors:** Anand R. Saran, Diana Kalinowska, Sangphil Oh, Ralf Janknecht, Luciano DiTacchio

A grant is missing from the Funding statement. This work was also supported by a National Institutes of Health grant (R01DK108088 to LD).

The full, amended Funding statement is included below:

National Institutes of Health grant number R01DK108088 to (LD). The funders had no role in study design, data collection, analysis, decision to publish, or preparation of this manuscript. This content is solely the responsibility of the authors and does not necessarily represent the official views of the National Institutes of Health. University of Kansas Medical Center Lied Pilot Project Grant FY2014 http://www.kumc.edu/kumcri/sponsored-programs-administration/internal-funding-opportunities/lied-basic-science-grant-program.html to LD. The funder had no role in study design, data collection and analysis, decision to publish, or preparation of the manuscript. University of Kansas Medical Center KINBRE Bridging Grant FY2015-16 to LD. The funder had no role in study design, data collection and analysis, decision to publish, or preparation of the manuscript. National Institute of General Medical Sciences https://www.nigms.nih.gov/Pages/default.aspx (grant number P20GM103549 and P30GM118247) to KUMC Department of Pharmacology, Toxicology, and Therapeutics. The funder had no role in study design, data collection and analysis, decision to publish, or preparation of the manuscript.
